# Medical Statistics

**Published:** 1838-01

**Authors:** 


### MEDICAL STATISTICS.

### *On the Prevalence and Mortality of Cholera in Venice, during the Years* 1835, 1836.

[THIS paper is drawn up from authentic documents, and the object of it is to prove, by statistical returns, that the cholera is not a formidable disease to all, but only to certain classes, whom he urges to apply for relief at the hospitals, instead of trusting to treatment at home. We give a summary of the results.]

TABLE I.—*Summary of* 286 *Cases of Cholera, occurring in a Population of* 9554 *Persons in three adjoining Parishes, from Oct.* 20, 1835, *till July,* 1836.

| | | | At home. | | In hospital. | | Class. | | | | | |
|---|---|---|---|---|---|---|---|---|---|---|---|---|
| Cases | Males | Females | Died | Cured | Died | Cured | i. | ii. | iii. | iv. | v. | Class not given |
| 286 | 150 | 136 | 122 | 58 | 62 | 43 | 60 | 55 | 25 | 55 | 14 | 90. |

The number of patients, compared to the population, is 1 in 33; exactly three per cent.

Of the three parishes, one contained none but persons of the very poorest class; of another about half, and of the third above half, possessed the means of support.

The proportion of cholera patients in the poorest to those in the wealthiest parishes in Venice, is 100 to 15.

The cures in the hospitals were to those at home as 70.5 per cent. to 47 per cent., or as 1.5 to 1.

The relative proportion of deaths in the two sexes, (males 98, females 86,) is very near the proportion of the cases: males 150, females 136.

The patients are nearly all comprised in the five classes of the table; the well-educated and respectable (*regolati*) being too few to constitute an exception.

The occupations of the patients appear to have had considerable influence in the production of the disease, particularly among the washerwomen, who, from the facts collected by the author, became affected in many instances from washing the clothes of the sick. Of washerwomen, there were 31; servants, 21; labourers, 34; masons, 13; cooks, 14; gondoliers, 8: almost all of them of the lowest order.

The persons included in the five classes of the table are the following:—1. Drunkards with wine or spirits, persons irregular in habits and diet. 2. Careless at the first appearance of disease, or who used improper remedies. (Two-fifths of the patients belonged to these two classes.) 3. Using bad food. 4. Affected with chronic complaints. 5. Bilious persons, the fearful, the weaker aged, the exposed to extremes of temperature.

It is a most remarkable circumstance that, of 2500 Jews inhabiting Venice, not one was attacked. The author attributes this immunity to their strict observance of their religious rites and ordinances, to their careful attention to their diet, and to their habit of retiring within their houses in the evening. [The Polish Jews suffered far more from cholera than the Christians in 1831, so that whole villages were almost depopulated; and this was attributed to their excessively dirty habits.]

TABLE II.—*Numerical Account of Cholera Patients treated in the Hospitals, from October* 9*th,* 1835, *when the Disease broke out, till December* 20*th, when the Hospitals were closed.*

| Men. | | | Per cent. | | | | | Total. | | | |
|---|---|---|---|---|---|---|---|---|---|---|---|
| Cases | Cured | Died | of deaths | Women | Cured | Died | per Cent. | Cases | Cured | Died | perCent. |
| 71 | 40 | 31 | $43\frac{96}{100}$ | 110 | 39 | 71 | $64\frac{54}{100}$ | 181 | 79 | 102 | $56\frac{35}{100}$ |

[If we take Venice as the average for Italy, the mortality by cholera in that country is a little higher than in Scotland, and lower than in France; the average deaths, from the official returns, being in England one-third, in Scotland one-half, in France two-thirds, of persons attacked.]

Table iii.—*Summary of Cholera Patients treated in the Hospitals, from March* 31*st, to June* 30*th*, 1836.

| Admitted. | | Cured. | | Dead. | | Under treatment. | | |
|---|---|---|---|---|---|---|---|---|
| Males | Females | M. | F. | M. | F. | M. | F. | Mortality per Ct. |
| 444 | 355 | 170 | 153 | 256 | 189 | 18 | 13 | 55.69. |
| Total, 799 | | 323 | | 445 | | 31 | | |

*Appendice al Bullettino delle Scienze Mediche di Bologna.* Fasc. 2. 1836.

---

### *Statistics of Cholera in Naples.*

The cholera in Naples, as elsewhere, exhibited three well-marked stages, in its commencement, increase, and decline. The first period lasted from the 2d till the 23d of October, when the weather was very tempestuous; and the number of cases and deaths increased rapidly for a month, when, about November 24th, the disease began to decline, and continued to decrease to the end of December, at which time it had almost entirely ceased.

| | Cases. | | Deaths. |
|---|---|---|---|
| First period, (commencement,) from Oct. 2d to 23d ........ | 210 | .. | 126 |
| Second period, (increase,) from Oct. 24th to Nov. 23d ...... | 6837 | .. | 3620 |
| Third period, (decline,) from Nov. 24th to Dec. 31st......... | 2184 | .. | 1314 |
| Total........... | 9231 | .. | 5060 |

The military are not included in this account: their numbers were, cases 359, deaths 135, which make the whole number of cases 9590, and deaths 5195; the cures were 4090, the remaining 305 being still under care.

| Quarters of the City. | Population | Cases. Total | Cases. Per Ct. | Deaths. Total | Deaths. Per Ct. | Locality. | Inhabitants. |
|---|---|---|---|---|---|---|---|
| Porto ............... | 36715 | 2410 | 6.57 | 1418 | 3.36 | Damp, unhealthy. | Very poor. |
| Pendino ............ | 31086 | 1179 | 3.79 | 551 | 1.77 | Ditto ditto | Ditto. |
| Mercato ............ | 51551 | 1821 | 3.53 | 977 | 1.89 | Ditto ditto | Ditto. |
| Chiaza ............... | 27606 | 641 | 2.32 | 421 | 1.50 | Rather better. | Better. |
| S. Carlo all' Arena | 21209 | 492 | 2.32 | 248 | 1.17 | Same as Stella. | Contains the poor-house |
| Vicaria.............. | 39653 | 755 | 1.90 | 386 | 0.97 | As Chiaza. | Better. |
| S. Ferdinando...... | 31204 | 575 | 1.84 | 261 | 0.83 | | |
| S. Guiseppe ........ | 19608 | 323 | 1.65 | 212 | 1.08 | Higher and healthier. | Better off. |
| S. Lorenzo ......... | 11191 | 168 | 1.50 | 105 | 0.94 | | |
| Montecalvorio .... | 31154 | 409 | 1.31 | 215 | 0.69 | On high ground, at a distance from the sea. | Wealthy. |
| Stella ............... | 23278 | 196 | 0.84 | 127 | 0.54 | | |
| Avvocata............ | 33028 | 262 | 0.79 | 145 | 0.43 | | |
| Citta Intera......... | 357283 | 9231 | 2.58 | 5066 | 1.41 | | |

The great majority of the persons attacked were the poor, the over-worked, the dirty, the depraved, the intemperate, and those who were exposed to the changes of weather. There were very few persons of property who suffered, except those who lived in unhealthy situations, or whose habits were such as above.

Many pregnant women took the disease, which was generally fatal to them.

*Returns from the Hospitals and Provincial Districts.*

| HOSPITALS. | Cases. | Deaths. | Cured. | Under Care. |
|---|---|---|---|---|
| Consolazione ...... | 819 | 515 | 276 | 27 |
| Sa. Maria de Loreto | 902 | 487 | 415 | |
| Total......... | 1721 | 1002 | 691 | |
| DISTRICTS. | | | | |
| Castellamare ...... | 1360 | 619 | 741 | |
| Cavoria ............ | 116 | 61 | 55 | |
| Pozzuoli ............ | 104 | 44 | 60 | |
| Total...... .. | 1580 | 724 | 856 | |

During January, 1837, 119 cases occurred, of whom 96 died; and 16 among the soldiers, of whom two died; which, added to the previous totals of 9590 cases and 5195 deaths, make a grand total of 9725 cases and 5293 deaths.

*Fil. Sebez. January,* 1837.

---

*Sydney Dispensary, New South Wales.*

[THE following short document possesses some interest in itself, as indicating the amount and relative prevalence of diseases in a climate of which we as yet possess scarcely any medico-statistical details: we confess, however, that it is more interesting to us as a testimonial of the progress of our science—ever the handmaid of benevolence,—in the new nations of the earth.]

"The Committee of the Sydney Dispensary have published their Report for 1836, and we are pleased to see in it an approximation to the usages of such Institutions in Britain, by which alphabetical lists of the diseases treated during the year are published for general information. This is a new feature in Colonial Reports of this kind, and will prove interesting to science and the colonists. Inflammatory complaints, it will be perceived, are more frequent than those arising from other causes. As a document of value, we present to our readers the following list of diseases without abridgment:—

Alphabetical list of the diseases treated at the Sydney Dispensary, from 1st May, 1836, to 30th April, 1837:—Abortion 1, Abscess 4, Amaurosis 1, Amenorrhœa 2, Aneurism 1, Aphthæ 1, Apoplexy 1, Asthma 4, Biliary derangement 7, Bubo 2, Burns and Scalds 12, Cancer of the Lip 1, ditto of the Mamma 2, Carbuncle 1, Cholera 1, Chorea 1, Colic 3, ditto Painters 2, Constipation 7, Consumption 9, Contusions 33, Cramp 1, Croup 2, Cutaneous diseases 14, Delirium Tremens 1, Dentition 4, Diarrhœa 20, Disease of the Heart 3, ditto of the Hip Joint 2, ditto of the Knee Joint 1, ditto of the Spine 1, Dislocations 3, Dropsy of the Belly 5, ditto of the Brain 1, ditto general 10, ditto of the Knee Joint 1, Dysentery 22, Dyspepsia 5, Erysipelas 9, Fever, continued 6, ditto, intermittent 1, Fistula in Perineo 2, Fracture of the Arm 3, ditto of the Clavicle 1, ditto of the Leg 3, ditto of the Ribs 1, Gangrene 1, Gastrodynia 4, Gonnorrhœa 12, Gout 1, Headach 6, Hemoptysis 1, Hemorrhoids 6, Hernia 6; Ileus 2, Imperforate Nostril 1, Inflammation, Bowels 5, ditto Bronchia 19, ditto Ear 1, ditto Eyes 38, ditto Liver 7, ditto Lungs 3, ditto Pericardium 3, ditto Periosteum 1, ditto Peritoneum 2, ditto Pleura 6, ditto Testes 6, ditto Throat 4, Influenza 27, Insanity 1, Jaundice 2, Leucorrhœa 4, Marasmus 5, Menorrhagia 4, Mercurial Disease 5, Necrosis 2, Palsy 9, Paraphymosis 1, Prolapsus Uteri 2, Rheumatism, Acute 7, ditto Chronic 32, Scurvy 2, Scrofula 7, Sprain 1, Strangury 7, Stricture 1, Syphilis 34, Toothach 1, Tumours 1, Ulcers 22, Varicose Veins 3, Vertigo 3, Worms 12, Wounds 12. Total number of cases, 560—of these nearly one hundred were attended at their own houses.

*The Sydney Herald. May* 29, 1837.

*Contributions to Statistics of Diseases.* By Dr. Caster.

The following table is not only interesting, as showing the proportions in which various diseases and malformations occur in France, but as affording an additional proof that those phenomena of nature which appear the most accidental,—e. g. the existence of hernia, &c.—are dependent on immutable laws, the existence of which the science of physico-medical statistics has yet to prove and to establish. The following table shows the number of young men who, in France, in the years 1831, 32, 33, were returned, under the recruiting system, and the diseases or defects on account of which they were returned.

| | 1831. | 1832. | 1833. |
|---|---|---|---|
| Deficiency of fingers . . | 752 | 647 | 743 |
| Deficiency of teeth . . | 1,304 | 1,243 | 1,392 |
| Deficiency of other limbs . . | 1,605 | 1,530 | 1,580 |
| Deafness and dumbness . . | 830 | 736 | 725 |
| Swellings of the glands of the neck, | 1,125 | 1,231 | 1,298 |
| Lameness . . . . . | 949 | 912 | 1,049 |
| Other "deformities" . . | 8,000 | 7,630 | 8,394 |
| Diseased bones . . . | 782 | 617 | 667 |
| Myopia . . . . . | 948 | 891 | 920 |
| Diseases of the eye . . . | 1,726 | 1,714 | 1,839 |
| Itch . . . . . . | 11 | 10 | 10 |
| Scald-head . . . . . | 749 | 800 | 794 |
| Tetters . . . . . | 57 | 19 | 29 |
| Other skin diseases . . . | 937 | 983 | 895 |
| Scrofula . . . . . | 1,730 | 1,539 | 1,272 |
| Diseases of the chest . . | 561 | 423 | 859 |
| Hernia . . . . . | 4,044 | 3,579 | 4,222 |
| Epilepsy . . . . . | 463 | 367 | 342 |
| Other diseases, not yet mentioned, | 9,168 | 9,058 | 10,286 |
| Weakness . . . . . | 11,783 | 9,979 | 11,259 |
| Too small stature . . . | 15,935 | 14,962 | 15,078 |

The regularity of the occurrence of diseases is here very remarkable. The only irregularity deserving notice is under the class "Tetters;" and the arrangement of diseases under this head would, of course, depend much upon the different opinions of the medical men. Any one accustomed to inquiries of this kind will readily grant that the slight disparities observable in the above list would appear much less, if the table included a period of from twenty to thirty years, instead of but three years.

*Wochenschrift für die gesammte Heilkunde.* No. 51. 1836.

